# A Catastrophic Biodiversity Loss in the Environment Is Being Replicated on the Skin Microbiome: Is This a Major Contributor to the Chronic Disease Epidemic?

**DOI:** 10.3390/microorganisms11112784

**Published:** 2023-11-16

**Authors:** Christopher Wallen-Russell, Nancy Pearlman, Samuel Wallen-Russell, Dragos Cretoiu, Dana Claudia Thompson, Silviu Cristian Voinea

**Affiliations:** 1 The Skin Microbiome School, Pavane Research Centre, Reading RG1 4QD, UK; kit@microbiomeschool.com (C.W.-R.);; 2 Ecology Center of Southern California, Los Angeles, CA 90035, USA; nancysuepearlman@aol.com; 3 Department of Medical Genetics, Carol Davila University of Medicine and Pharmacy, 020021 Bucharest, Romania; 4 Fetal Medicine Excellence Research Center, Alessandrescu-Rusescu National Institute for Mother and Child Health, 011062 Bucharest, Romania; 5 Department of Surgical Oncology, Prof. Dr. Al. Trestioreanu Oncology Institute, Carol Davila University of Medicine and Pharmacy, 022328 Bucharest, Romania

**Keywords:** catastrophic biodiversity loss, skin microbiome, biodiversity, microbiome, chronic disease epidemic, skin allergy epidemic, butterfly effect, entropy, chaos theory

## Abstract

There has been a catastrophic loss of biodiversity in ecosystems across the world. A similar crisis has been observed in the human gut microbiome, which has been linked to “all human diseases affecting westernized countries”. This is of great importance because chronic diseases are the leading cause of death worldwide and make up 90% of America’s healthcare costs. Disease development is complex and multifactorial, but there is one part of the body’s interlinked ecosystem that is often overlooked in discussions about whole-body health, and that is the skin microbiome. This is despite it being a crucial part of the immune, endocrine, and nervous systems and being continuously exposed to environmental stressors. Here we show that a parallel biodiversity loss of 30–84% has occurred on the skin of people in the developed world compared to our ancestors. Research has shown that dysbiosis of the skin microbiome has been linked to many common skin diseases and, more recently, that it could even play an active role in the development of a growing number of whole-body health problems, such as food allergies, asthma, cardiovascular diseases, and Parkinson’s, traditionally thought unrelated to the skin. Damaged skin is now known to induce systemic inflammation, which is involved in many chronic diseases. We highlight that biodiversity loss is not only a common finding in dysbiotic ecosystems but also a type of dysbiosis. As a result, we make the case that biodiversity loss in the skin microbiome is a major contributor to the chronic disease epidemic. The link between biodiversity loss and dysbiosis forms the basis of this paper’s focus on the subject. The key to understanding why biodiversity loss creates an unhealthy system could be highlighted by complex physics. We introduce entropy to help understand why biodiversity has been linked with ecosystem health and stability. Meanwhile, we also introduce ecosystems as being governed by “non-linear physics” principles—including chaos theory—which suggests that every individual part of any system is intrinsically linked and implies any disruption to a small part of the system (skin) could have a significant and unknown effect on overall system health (whole-body health). Recognizing the link between ecosystem health and human health allows us to understand how crucial it could be to maintain biodiversity across systems everywhere, from the macro-environment we inhabit right down to our body’s microbiome. Further, in-depth research is needed so we can aid in the treatment of chronic diseases and potentially change how we think about our health. With millions of people currently suffering, research to help mitigate the crisis is of vital importance.

## 1. Introduction

“Catastrophic biodiversity loss” is a phrase used by ecologists like David Attenborough to describe what is happening in macro-ecosystems across the world due to human intervention. The dire consequences of it are well documented [1]. But is a parallel biodiversity loss happening right here in our own bodies? And what is the impact on the developed world’s health?

The rapid growth of allergies and chronic diseases in the Western world, named the “chronic disease epidemic”, is now attributed to 90% of healthcare costs in the USA [2,3] and 74% of global mortalities [4]. The exact causes remain formally unexplained [5], but growing evidence suggests a causal link between the loss of microbial diversity in the gut microbiome and a plethora of health issues [6,7]. This is thought to be influenced by modern lifestyle factors such as the Western diet and antibiotics in food [7,8].

Previous instrumental work used aspects of macro-ecology to describe how a significant loss of microbial diversity in the gut microbiome is associated with “most of the human diseases affecting westernized countries” [7]. However, although the majority of the body’s bacteria reside inside the gastrointestinal tract, the human microbiome is composed of much more than just the gut [9]. Microbial ecosystems are present in other locations all over the body, including on the skin, in the nasal passage, the lungs, the throat, and the vagina [10].

The skin, with its estimated 10^12^ inhabiting bacteria, represents the second most populous site of the human body [9]. As the body’s largest organ and the first line of defense against environmental stressors, it is crucial for maintaining an effective immune system [11] and is a vital part of the nervous and endocrine systems [12,13]. Despite this, the skin microbiome and its influence on whole-body health are less frequently discussed in comparison to the gut.

In this paper, we will be focusing on the area of biodiversity loss as a potential marker for disease. We note that there are other possible markers, including site-specific abundances of microbial species and strains within the microbiome, but an in-depth evaluation of these is not within the scope of this paper.

We also note that chronic disease development is likely complex and multifactorial. Despite a genetic predisposition playing a role, the ultimate trigger is unknown in many cases and could be a combination of multiple other factors, such as a dysbiotic microbiome as discussed here, diet, lifestyle, stress, age, access to healthcare, and exposure to chemical pollutants in the environment [14,15].

## 2. Biodiversity Loss and the Skin Microbiome

### 2.1. Biodiversity Loss in Global Ecosystems

In global macro-ecosystems, ecologists are concerned that humans are causing a sixth mass extinction event occurring at a pace far exceeding background rates [16]. The Living Planet Index reports a 69% average decline in wildlife populations since 1970 [17], with predictions suggesting that a further 1 million animal and plant species may face extinction within decades [18].

Biodiversity loss is mainly driven by habitat loss and fragmentation [1]. A testament to this is the loss of 90% of wetlands since 1700 [19], accompanied by an exponential rise in human land use, expanding from 10% to 50% of the Earth’s landmass [20]. In a little over a century, forest areas equivalent to the size of the United States have vanished, leaving less than 3% of Earth’s total land with ecological integrity while exerting high pressure on 58% of ecosystems [21].

The world’s oceans are also under threat. Home to around 25% of fish species and other life forms, the world lost 14% of its coral [22] between 2009 and 2018. Other studies say coral reefs may become extinct within our lifetimes [23], and one-third of marine mammals, sharks, shark relatives, and reef-forming coral are also threatened with extinction [17].

### 2.2. The Skin Microbiome, Biodiversity, and Dysbiosis

In parallel to our natural environment, the human body is a thriving ecosystem host to trillions of living organisms [24]. One study suggests that only 43% of the human cell count belongs to the individual, while the rest consists of microbes [9]. This ecosystem is so important that one author believes that “understanding the structure and function of the human symbiont communities might become the first great breakthrough of twenty-first century medicine” [25]. Thousands of research papers and probiotic health proposals have propelled the human gut microbiome and its influence on whole-body health to almost mainstream levels of awareness [26,27]. And rightly so, in addition to being an essential part of the body’s complex, interlinked immune system, the gut microbiome also plays a key role in water absorption and nutrient metabolism [7].

Skin microbiome papers are outnumbered by their gut microbiome counterparts at a ratio of 10 to 1 in the PubMed database. And while in recent years it appears that its role in skin health and disease is becoming appreciated, we are only just beginning to realize the skin microbiome’s potential impact on whole-body health. For example, the latest studies are starting to shed more light on the gut-skin axis, its involvement in human immune responses, and the associated pathologies [28,29,30,31]. It is believed that allergic sensitization through damaged skin is a main factor in food allergy development [32,33].

The human skin microbiome consists of a variety of bacteria, fungi, and viruses, which play an important part in immune system training, external pathogen protection, and natural product metabolism [34,35,36]. In recent years, emerging novel technologies such as shotgun metagenomic sequencing have enabled scientists to characterize these microorganisms in detail, according to the skin site, the age of the individuals, the human habitat, and their variability throughout time [37].

The intrinsic factor that contributed to the largest compositional differences observed in the skin microbiota has been found to be the skin site microenvironment. A large variation was found between dry, oily, and sebaceous sites on the skin [11,38,39]. For example, *Cutibacterium* species were more abundant in sebaceous sites such as the manubrium, the face, and the back, while *Corynebacterium* and *Staphylococcus* species that flourish in humid environments were more dominant in moist regions like the axillary vault, the groin, and the toe web [11,37].

Altered microbial signatures have also been found more frequently in disease states compared to healthy skin, where the delicate balance of the ecosystem has been disturbed, often becoming harmful to the host. This is referred to by the umbrella term “dysbiosis”.

When talking about dysbiosis, it is important to pin down exactly what it is because the use of the term immediately elicits the view of a damaged system. It is defined as “an imbalance in bacterial composition, changes in bacterial metabolic activities, or changes in bacterial distribution” [40]. Dysbiosis can be categorized into three main types: loss of beneficial organisms, excessive growth of potentially harmful organisms, and loss of overall microbial diversity [40]. It is important to note that in most cases, these types of dysbiosis happen at the same time.

The important question is: what constitutes a sufficient loss of beneficial organisms or excessive growth of potentially harmful organisms? This will have to be considered on a case-by-case basis, with a specific understanding of each system and how to identify a healthy or diseased system. Providing a definitive and global method to measure dysbiosis by using overgrowth or loss of specific bacteria is an ongoing challenge that is made difficult due to the aforementioned large variation between different sites and individuals [41,42,43]. For example, a dysbiotic skin microbiome in atopic dermatitis displayed global and body-site-dependent variations [44], and despite years of research, no single pathogen has been identified as the cause of psoriasis [45].

The difficulty in finding one exact composition for health and disease is exemplified in the gut microbiome, where “no gold standard exists to determine the presence or extent of a given imbalance or disturbance” [46]. This is because researchers still do not have a clear definition to identify a healthy gut microbiome [41].

A commonly used measure of dysbiosis is alpha and beta diversity [46], which makes sense because biodiversity loss is one of the types of dysbiosis. This paper focuses on biodiversity loss as a possible marker for disease due to its relation to dysbiosis.

When a system is in dysbiosis, it is probable, but not certain, that it has experienced biodiversity loss. More research is needed to understand the instances in which there is an increase in diversity during dysbiosis. The important point here is that dysbiosis of the microbiome has been implicated in many diseases across the board, indicating poor health [46].

In global macro-ecosystems, it is widely accepted that an increase in biodiversity corresponds to increased health and stability, with both experimental and theoretical evidence used to highlight this point [47,48,49,50]. This pattern also appeared in the gut microbiome, where many authors relate diversity to host health [7,51,52], and reduced biodiversity is linked to a vast number of human diseases [7,53]. Crucially, a loss of microbial biodiversity is also thought to be “the most common marker in intestinal dysbiosis” [7]. On aggregate, and acknowledging that there are some research cases where disease states appear to show an increase in biodiversity, this appears to be replicated in the skin microbiome (see Section 3.2). Previous data shows that healthy skin is inhabited by a much more biodiverse ecosystem than unhealthy or diseased skin [54,55]. One author stated “The biodiversity of the skin microbial ecosystem can be directly linked to the skin’s overall health, since skin diseases, such as atopic dermatitis and psoriasis, are often associated with dysbiosis” [56]. For these reasons, multiple authors have spoken of the need for future skin ailment solutions to increase the biodiversity of the skin microbiome [57,58,59].

It is important to note that one person’s healthy microbiome will be different from another’s, and it will differ across body sites. Therefore, we are careful to talk about biodiversity “increase” or “decrease” throughout this paper instead of describing standalone figures such as “high” or “low”.

### 2.3. Are We Seeing a Loss of Biodiversity in the Human Skin Microbiome, Just like in the Environment and Our Gut?

The healthiest intestinal tracts ever recorded were found in infants dwelling in rural Burkina Faso [60,61]. They lacked western gut problems and displayed large differences in composition, coupled with extremely elevated biodiversity compared to urban, city-dwelling children. This decreased biodiversity of the gut microbiome of people in industrialized western countries was later supported by multiple other studies [7,62,63,64]. The Hadza tribe from northern Tanzania was found to have an average of 730 species of gut microbes per person, compared to 277 and 436 for Californians and Nepalese farmers, respectively [65]. It is now thought that modern humans have lost 50% of the gut microbiota of our primate ancestors [66]. This “mass extinction”, mirroring macro-ecosystems, has been attributed to exposure to Western world practices such as diet and the overuse of antibiotics [7,51,65,67].

The same phenomenon described for the digestive system and global ecosystems was found to have also occurred on the skin of western humans; isolated tribespeople, called the Yanomami people, with negligible documented contact with the outside world, displayed unprecedented levels of biodiversity in their skin microbiome [68]. Furthermore, there was no evidence of modern skin problems such as acne and eczema [34,42], despite the former affecting 79–95% of adolescents [69]. Similar findings were reported in other studies, which found acne vulgaris to be a condition affecting primarily developed countries but not people living in rural Papua New Guinea, Paraguay, or Brazil [69,70]. Due to the Yanomami tribe living in relative isolation for over 11,000 years since their ancestors arrived in South America, they could possess skin microbiomes that more closely resemble those of our ancient human ancestors.

When other cultures were analyzed, this phenomenon was not an anomaly. A study that tested five areas at the same latitude in the Amazon rainforest found that the microbial biodiversity of the skin microbiome decreased with increased urbanization, mirroring research on the gut [71]. Further work reported significantly increased biodiversity of the skin microbiome in indigenous people, farmers, and those living in rural settings compared to their urban counterparts [72]. In addition, a paper reported interesting findings on a group of Amerindians living in the Venezuelan Amazon, called the Guahibo people, who lived in rural settlements without running water or electricity; half of the group still possessed much more biodiverse microbiomes than those in the developed nations [73]. However, this was the only study on these isolated people, with some nuance to the findings. The Amerindians could be categorized into two groups with distinct bacterial communities: the first one had much higher biodiversity than Americans, and the second one had similar biodiversity but was dominated by *Staphylococcus* and not *Cutibacterium*. A possible explanation is that some level of *Staphylococcus* infection could have been more severe in one group, which lowered the biodiversity [74].

**Figure 1:**
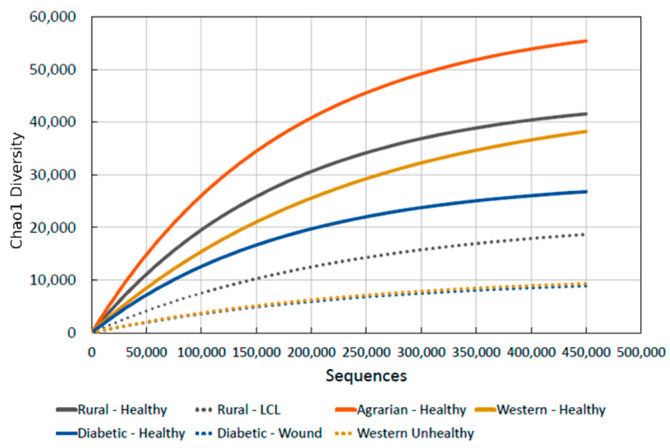
A graph showing the comparative biodiversity benchmarks for different skin health states taken from previous work [55]. For an in-depth explanation and background on each, please refer to the original study. The skin of individuals in basic settlements with limited urbanized practices is reduced in biodiversity by 25% compared to Amerindians, and even individuals with the healthiest skin in Western environments, labeled “Western-Healthy,” exhibit a 30% reduction in biodiversity compared to the “Agrarian-Healthy” skin of Amerindians. The decrease falls further to 51% for individuals with diabetes but without skin lesions, 64% for diabetic individuals with skin wounds, and an alarming 84% reduction in biodiversity for individuals with skin diseases in the Western world.

**Figure 2:**
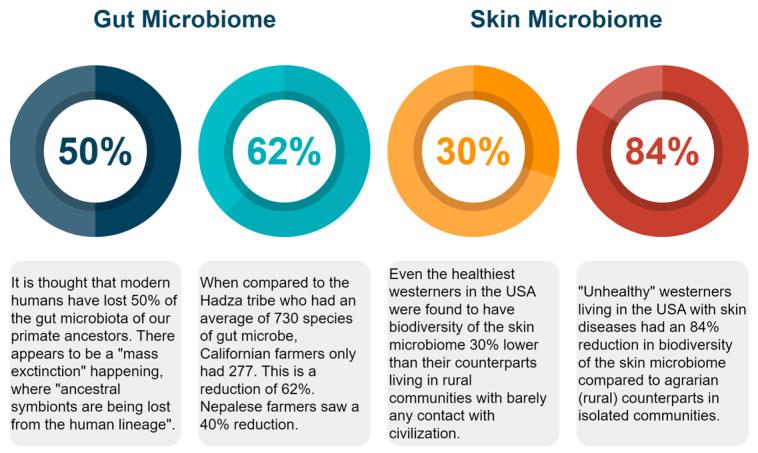
A comparison of biodiversity loss as percentages for the gut and skin microbiome.

In our previous work, we quantified skin microbiome biodiversity across various health states [55]. The comparison graph in Figure 1 demonstrates a progressive decrease in biodiversity from the “caveman” skin of Amerindians down to the lowest seen in people with skin ailments in developed Western countries. This shows a skin biodiversity reduction of 30% to 84% in the developed world. Figure 2 summarizes gut and skin biodiversity loss.

### 2.4. What Is Causing the Biodiversity Loss in the Skin Microbiome?

Here we summarize the research to provide an overview while noting that it is beyond the remit of this paper to come to a conclusion on the main contenders. Further research needs to be done to answer this, which may be an important part of tackling the chronic disease epidemic. This section is included to highlight how, as a phenomenon only observed on the skin of people living urban, modern lives, there must be external factors in our environment which are the main causes.

**Figure 3:**
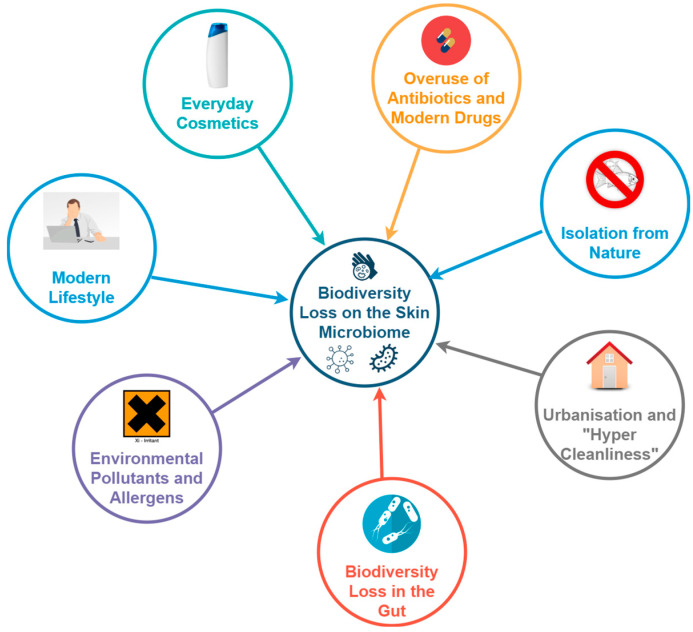
Factors thought to be contributing to the large reduction in biodiversity in the skin microbiome of people in the urbanized, developed world.

The exposure of the body to 21st-century soaps, cosmetics, pollution [75], medicine, drugs, detergents, antibiotics, and synthetic chemicals in cleaning products appears to have caused significant microbiome alterations [36,38,76,77,78,79,80,81,82,83,84]. A modern lifestyle characterized by stress, poor diet, and indoor living isolated from nature is also thought to play a part. The skin’s role as the external barrier between the body and the environment suggests it could be even more susceptible to extrinsic factors than the gut. Figure 3 summarizes the factors listed below.

The advent of modern medicine and pharmaceutical interventions has brought about a remarkable transformation in global health, arguably unparalleled in human history. However, a new challenge has emerged, stemming from the excessive use of medications intended for acute illnesses but now being used for long-term management of chronic conditions. A common 21st-century example of this is the excessive use of antibiotics [11,43], which decreases microbiome biodiversity [85] and can induce dysbiosis on the skin [86]. Their indiscriminate approach to eliminating microbes [87] can render the skin vulnerable to pathogens that were previously warded off by a great proportion of resident and mutual bacteria [80,85].

Our hyper-sanitized indoor living environments and increasing isolation from nature [88] are associated with microbiome depletion and immune system malfunction (referred to as “dysregulation”) from reduced exposure to a diverse range of microbes. Such exposure is crucial for training an innate and adaptive immune system [89], and without this, our body becomes less effective at protecting against disease [90]. The biodiversity and hygiene hypotheses describe this issue [91,92]. One line of evidence for this is offered by “alpine altitude climate treatment” for eczema. The resultant significant increase in the skin’s microbial diversity [93] was said to be due to the urban living environment’s environmental pollutants, high aeroallergen count, altered UVB exposure [94], and reduced exposure to microbes, which are depleting the human microbiome [95].

Interestingly, only when indigenous people moved to industrialized cities did acne become a problem [96]. In addition, the number of chemicals and detergents found in houses rises rapidly with increased urbanization [71], which is negatively correlated with the biodiversity of the skin microbiome [71]. A more recent paper, summarizing its findings, suggested that “these products might account, at least in part, for the loss of diversity in the cutaneous bacterial communities in urban settings” [97]. Especially pertinent during 2020 and 2021 due to the SARS-CoV-2 global pandemic, regular exposure of the skin to disinfectants on surfaces or direct application to the skin can “induce hazardous skin conditions”, penetrate the skin, and disrupt its barrier functions [98].

Through the gut-skin axis, an altered immune response due to gut dysbiosis and loss of biodiversity is thought to play a role in the development of common skin diseases [31]. There could also be an association between gut problems and reduced biodiversity in the skin microbiome [28]. Thus, the gut microbiome’s reduced biodiversity in the western world should be mentioned.

Everyday cosmetics, often containing a substantial proportion of synthetic ingredients, have been implicated in microbiome alterations and associated health issues [76,99,100,101,102,103,104] and can strip the skin of its essential oils and bacteria [78,105]. One reason for this could be that they often have a pH of 5.5 or above, which can alter the skin’s natural pH, decrease biodiversity, dry out the skin, and cause skin irritation [106,107,108,109]. A study found cosmetics with a high synthetic chemical percentage had a pH of 6, compared to 4.5 for a 100% natural formulation [76]. In contrast, natural, healthy skin has a pH lower than 5 [110]. A skin pH that is too alkaline is thought to become less hostile to pathogenic microbes, disturbing the balance of the normal microflora [111] and leaving the skin prone to infection and disease [112]. Soaps have pH values of around 9.5–10.5, and a single wash can increase skin pH to 7.5 [100].

However, testing the effect of cosmetics on biodiversity directly has given mixed results; some studies show an increase or no change, suggesting the need for future research [113]. Molecules from cosmetic products persisted on the skin for weeks after showering, which altered the skin microbiome [114], and detergents correlated with reduced skin biodiversity [71]. Furthermore, excessive cosmetic use has been implicated in the triggering or exacerbation of various skin ailments such as rosacea [115], eczema, allergies [116], and irritation [117,118]. This effect extends beyond the skin, where cosmetic use has been linked to an elevated risk of breast cancer and a rise in asthma prevalence [119,120,121]. Ingredients in these products, like methylisothiazolinone (MI) [122], still found in natural-labeled cosmetics in 2018 [76], and parabens, are linked to skin allergies, microbiome disruption, hormonal imbalance, and reduced biodiversity [123,124,125,126]. Additionally, synthetic fragrances can trigger allergies, migraines, and hormone disruption [127,128].

## 3. Is Biodiversity Loss on the Skin Involved in the Chronic Disease Epidemic?

### 3.1. The Chronic Disease Epidemic

A chronic condition is defined as “a physical or mental health condition that lasts more than one year and causes functional restrictions or requires ongoing monitoring or treatment” [129]. The 1950s saw a distinct shift in the dominant health problems in the USA; the previously more common acute diseases were replaced by chronic diseases [5]. Today, 50% of Americans are living with at least one chronic disease, which accounts for 86% of all healthcare costs [5] and costs an estimated $3.7 trillion annually [130]. Between 2000 and 2020, chronic disease prevalence grew by around 28 million people [5].

**Figure 4:**
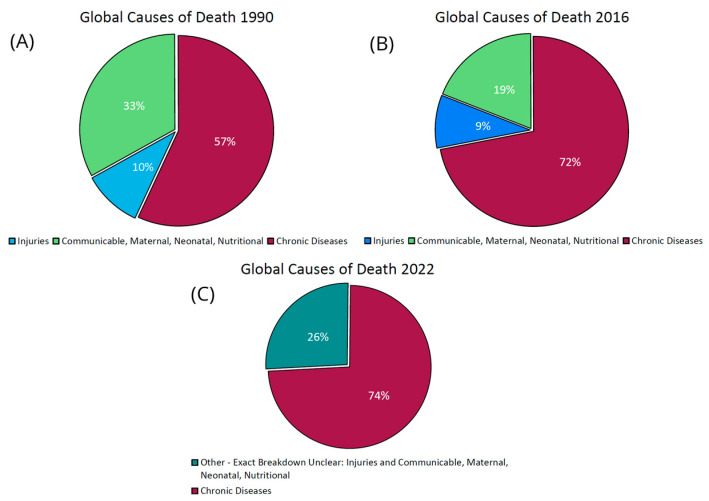
Global causes of death from 1990 to 2016. (**A**) shows that 57% of global deaths in 1990 were from chronic or “communicable” diseases, compared to 33% for “communicable”, maternal, neonatal, and nutritional diseases, and 10% for injuries. (**B**) shows that these percentages for the same categories in 2016 were 72%, 19%, and 9%, respectively. (**C**) shows that these percentages for the same categories in 2016 were 74% and 26% for the combined last two categories, as the data was unclear. The data to make (**A**,**B**) was taken from Anderson and Durstine, 2019 [139], and (**C**) from the 2022 report from the World Health Organization (WHO) called “Noncommunicable Diseases: Progress Monitor 2022” [4].

**Figure 5:**
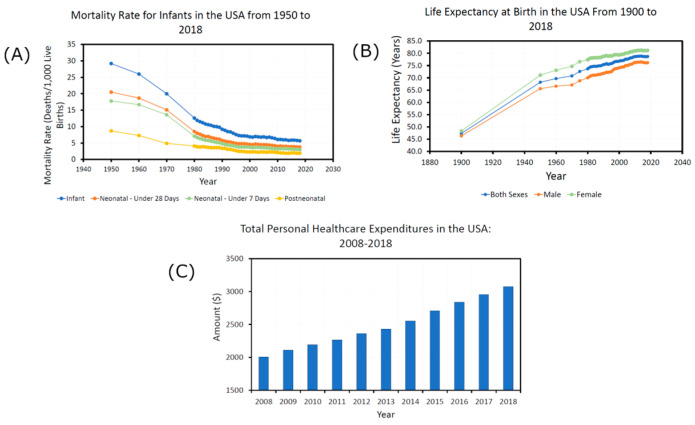
Health statistics graphs for the USA created from data collated in the report “Health, United States 2019” [140]. Graph (**A**) shows the infant mortality rate in the USA, measured in deaths per one thousand infants. It decreased from 29.2% in 1950 to 5.7% in 2018. Graph (**B**) shows the life expectancy at birth for people living in the USA, split into male and female numbers, as well as an overall average. It increased from 47.3% in 1900 to 78.7% in 2018. Graph (**C**) shows the total healthcare expenditure per person in the USA, which increased from $2009 in 2008 to $3076 in 2018.

This was indicative of a global trend; the prevalence of chronic human diseases in the developed world has continued to increase at an alarming rate throughout the 20th century and the start of the 21st [7,131,132]. As Figure 4 shows, it is now the leading cause of death, rising from 57% of global mortalities in 1990 [133] to 74% in 2022 [4]. This includes immune-related conditions such as allergies and multiple sclerosis and metabolic disorders such as type 2 diabetes and obesity [134,135,136,137]. Initially limited to Western nations, the occurrence of chronic diseases has spread to developing nations with the adoption of Western lifestyles [7,138]. This has all occurred in an age where decades have been added to the average human life expectancy, mortality rates have decreased by a factor of five between 1950 and 2018, and healthcare spending has increased rapidly—all signs of dramatic improvements in global health, as shown in Figure 5. However, the problem has become so severe that current estimates suggest that future generations may experience a decrease in life expectancy [139].

**Figure 6:**
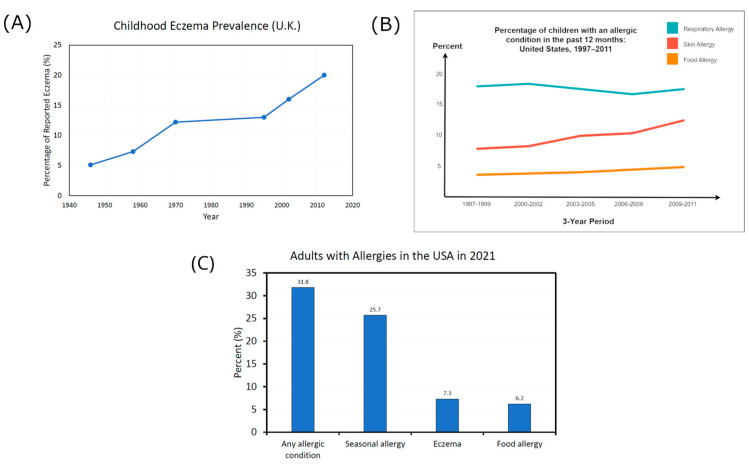
Graph (**A**) shows childhood reported eczema prevalence rates in the United Kingdom, which rose from 5.1% in 1946 to 20% in 2012. Data was taken from three sources [141,143,149]. Graph (**B**) shows the percentage of children aged 0–17 years who reported an allergic condition in the past 12 months from 1997–2011 in the USA. It shows a significant linear increase for food and skin allergies; however, skin allergies seem to be accelerating at a faster rate. Graph (**B**) was adapted from SOURCE: CDC/NCHS, Health Data Interactive, National Health Interview Survey, https://www.cdc.gov/nchs/data/databriefs/db121.pdf (accessed on 26 June 2023) [150]. Graph (**C**) shows the percentage of adults with a diagnosed seasonal allergy (25.7%), eczema (7.3%), food allergy (6.2%), or any allergic condition (31.8%) in the United States in 2021 [151]. The criteria for adults counted as having an allergic condition included being diagnosed with one or more of the three conditions shown in the graph. There is some overlap between the three conditions, and the estimated percentages are taken from interviews with a sample of the civilian population in America.

Contained within these statistics, the prevalence rate of allergic conditions and ailments of the skin has increased and even accelerated in recent years [54,78,141,142,143,144,145,146,147], leading to some calling it an “allergy epidemic” [148]. Figure 6A below shows how the UK eczema prevalence rates in children increased by around 400% from 1946 to 2011 [141,143,149]. An example of recent acceleration is shown in Figure 6B, where between 1997 and 2011, the prevalence of respiratory allergies stayed the same in American children, but food and skin allergies increased [150]. The USA National Health Interview Survey in 2021 found 31.8% of Americans had an allergic condition, including 7.3% with eczema [151] (Figure 6C). A further survey of eleven thousand adults from five major countries found 35.6% were living with allergies [152].

### 3.2. Skin Diseases Associated with Biodiversity Loss

Dysbiosis and biodiversity loss in the gut have been linked to a huge number of chronic health problems, including systemic diseases, and the prevailing belief is that they play an integral role in their development [6,7].

Just as observed in the gut microbiome, where a loss of microbial diversity was the most common finding in dysbiosis [7], it appears that reduced biodiversity is also a common observation in dysbiotic, diseased skin. There have been instances where papers reported an increase in diversity for certain diseases, which seems to go against the overall trend for the skin and across nature [153,154,155]. Discrepancies could be due to differences in body sites, sampling methods, diversity measuring methods, or large intra- and inter-personal variation in the skin microbiome [153,156,157]. Much more in-depth research into the skin microbiome is needed to gain a more definitive answer on the link between biodiversity and diseased skin.

In parallel to the gut, a similar dysbiosis and reduced biodiversity of the skin’s ecosystem have been observed in many skin problems, including acne [158], atopic dermatitis and eczema [159] (including a drastic reduction during flares [142,160]), rosacea [161], psoriasis [162,163], tinea pedis (athlete’s foot) [59], diabetic skin wounds [164], cutaneous leishmaniasis [165], hidradenitis suppurativa [57,166], and skin cancer (in pigs) [167].

However, this does not immediately mean that it is biodiversity loss that is causing the skin problems. Thus, in Section 3.3 and Section 3.4, we will ask two main questions. Firstly, is reduced biodiversity observed on the skin of people with more systemic diseases in areas other than the skin? Secondly, is biodiversity loss a cause or a symptom?

### 3.3. Is Biodiversity Loss in the Skin Microbiome Associated with Systemic Diseases?

While research is still in its infancy, there is emerging evidence highlighting links between biodiversity loss in the skin microbiome and systemic chronic health problems, not just those affecting the skin.

For example, investigations have found that the lower the diversity of the skin microbiome and the more unbalanced the distribution of species, the more intense the systemic lupus erythematosus symptoms experienced, and concluded that the associated dysbiosis could be involved in the disease’s pathogenesis [168]. Additionally, changes in the abundance of specific bacterial taxa, such as *Staphylococcus aureus* and *Staphylococcus epidermidis*, have been identified as potential markers for associated skin lesions, supporting their conclusion that addressing the shift in the skin microbiome could be a “therapeutic target” for systemic lupus erythematosus rather than exclusively a symptom.

Research into the gut-skin axis provides more evidence. When this axis is referred to, most of the focus is on how the gut affects the skin, not the other way around [31]. One of many examples is that resultant internal microbiome alterations due to antibiotic use during infancy are linked to an increased likelihood of atopic dermatitis development [7].

However, it is a two-way relationship. Exposure to certain foods through damaged skin and a disrupted barrier is thought to contribute to the onset of food allergies [169]. In addition, vitamin D production mechanisms in the skin due to UVB exposure were found to significantly alter the diversity of the gut microbiome, leading authors to suggest the “existence of a novel skin-gut axis that could be used to promote intestinal homeostasis and health” [94]. It could transpire that reduced biodiversity on the skin could also impact diminished biodiversity in the gut, creating a negative feedback loop that further impacts gut issues and more systemic problems [31].

Like food allergies, it is also thought that exposure and sensitization through the skin may be an important factor in the development of asthma [170,171]. The overlapping pathogenic mechanisms between atopic dermatitis and asthma could highlight this connection [172].

Skin ailments associated with reduced biodiversity often coexist in individuals with cardiovascular diseases, such as atherosclerosis and hypertension, and were traditionally considered symptomatic of the underlying issues [173]. However, recent evidence suggests that this may not merely be a surface manifestation but could indicate a significant role of the skin in regulating the cardiovascular system. Mice without certain proteins in their skin reacted drastically differently to low oxygen levels than healthy mice, significantly affecting the body’s ability to circulate blood—a key factor in the development of heart disease and stroke [174].

Reduced microbial diversity and microbiome imbalances have also been observed for people with obesity [28,175], diabetes [164] (despite a contrary finding on the feet [176]), Alzheimer’s and schizophrenia [177], Parkinson’s disease [178], systemic sclerosis [179], IBD [180], and cirrhosis [181]. There appear to be a minimum of 27 diseases associated with reduced biodiversity on the skin.

### 3.4. Biodiversity Loss: Cause or Symptom?

It is now emerging that biodiversity loss on the skin could play a causal role in chronic disease instead of merely being a symptom [182]. In the gut, decreased biodiversity precedes allergy onset [183,184,185] and triggers Crohn’s disease in mice [186]. On the skin, dysbiosis and reduced microbial diversity precede atopic dermatitis (AD) onset [187], play a critical role in its manifestation due to *S. aureus* overgrowth [182], predict the persistence of eczema throughout childhood [188], and lead to epidermal barrier defects and skin immune dysregulation, which drive AD pathogenesis [189]. Dysbiosis on the skin is now known to be a cause of skin inflammation in AD [190]. In addition, improvement of the AD condition was found to be directly related to an increase in the biodiversity of the skin microbiome and not a decrease in *S. aureus*, as suggested [6]. Similar findings were found by Kennedy et al. and Kong et al., who found that during AD treatment, an increase in microbial diversity was indicative of an elevated chance of remission [74,142].

Its implication in the pathogenesis of systemic diseases adds further corroborating evidence in support of this idea. Dysbiosis of the skin microbiome was hypothesized as an “essential mediator” in inducing autoimmune diseases due to its role in systemic lupus erythematosus pathogenesis, as the resultant overproduction of a type of protein is thought to facilitate colonization by *Staphylococcus aureus* [14].

In addition, an indicator that a patient might develop asthma is a history of early-onset and severe atopic dermatitis [172]. Researchers discovered what may be the cause of this: damaged skin cells can secrete substances into the bloodstream that can induce a heightened immune response [191]. Once the substance reaches the lungs, it can trigger an allergic inflammation seen in asthma.

As we have already introduced, it is also thought to be contributing to the development of systemic sclerosis, cardiovascular diseases such as atherosclerosis and hypertension, and food allergies [192].

The skin is even implicated in neurodegenerative disease development. Ecosystem imbalance and the resultant fungal infections increase the likelihood of Parkinson’s disease development [178], where skin inflammation and the existence of disease-associated proteins on the skin and in the central nervous system precede its onset [193,194]. A line of thought suggests that *Malassezia* infections, linked to dysbiosis on the skin, could contribute to Parkinson’s disease due to the fungi’s role in pro-inflammatory cytokine production, which could trigger neurodegeneration in the brain [195]. Peptides released by an overgrowth of commensal skin flora have been found to speed up the accumulation of proteins linked to the disease [196].

Further research is needed to establish clear causal relationships or determine whether reduced biodiversity is a symptom or a mixture of both. People with severe skin diseases often have systemic issues too [192]. Because the body is a complex, interlinked system, it is likely they influence each other.

### 3.5. Potential Mechanisms of Chronic Disease Development

A biodiverse skin microbiome has been shown to result in a more effective immune system due to its key role in training both its innate and adaptive branches [11,197,198,199]. It also forms the first line of defense against environmental stressors [200]. Consequently, skin microbiome dysbiosis induces immune system malfunction, which can weaken colonization resistance against disease-associated pathogenic microbes, allow the entry of toxins into the blood, lessen the effectiveness of memory immune cells, induce chronic inflammation, and play a role in allergic sensitization—all factors involved in the onset of diseases.

A weakened skin barrier is another effect associated with dysbiosis and biodiversity loss, which could also be involved in allergic sensitization through a weakened epidermal barrier for ailments such as asthma and food allergies [170]. Healthy skin is less likely to sensitize to substances it encounters, especially non-irritants [201]. However, when the skin is damaged and chronically inflamed, sensitization to non-irritants and weak allergens can occur due to an altered immune T cell response [202]. Disruption of the natural barrier function can also lead to the penetration of pathogens and environmental stressors, potentially contributing to systemic conditions due to infiltration of the bloodstream.

Many of the risk factors and mechanisms for chronic disease development overlap; for example, the immune and inflammatory mechanisms involved in psoriasis and other issues such as depression and cardiovascular disease [203,204]. It is extremely hard to say why a disease develops, but we can look for universal markers, one of which appears to be dysbiosis, or a loss of biodiversity. This could lead to a range of alterations to the skin, which may have cascading knock-on effects on the body. The underlying mechanisms connecting the skin microbiome to disease pathogenesis are undoubtedly complex and require further investigation, especially in an intricate ecological network containing millions of interacting components [205].

We have already introduced some disease-specific mechanisms, but broadly speaking, it appears that a major factor is the induction of chronic inflammation. It has now been proven that damaged skin can induce not just local but systemic inflammation [32,195], thought to be a key driver of diseases such as stroke, cancer, chronic kidney disease, eczema, autoimmune and neurodegenerative conditions, ischemic heart disease, and diabetes mellitus, which make up 50% of global deaths [206]. An imbalance and biodiversity loss on the skin alter the skin’s natural biochemical conditions [195] and allow proinflammatory chemicals to enter the blood stream, thickening arteries, enlarging the heart, and damaging tissues and other systemic organs often thought unrelated [192]. They could also deposit fungal matter in the central nervous system, leading to a reduction in cognitive function [193].

Healthy, biodiverse skin possesses inherent defense mechanisms, such as the secretion of substances like sebum and dermcidin, which exhibit innate antibiotic effects [207,208,209,210] and prevent microbial dispersal, thereby guarding against pathogenic microbial growth [79,110,211]. Disturbances in this balance and a decrease in biodiversity may compromise their effectiveness.

The amplification of bacterial infections and autoimmunity in lupus patients could point to a potential negative feedback loop between loss of biodiversity in the skin microbiome and chronic diseases [212]. Lastly, the gut-skin axis, being a part of the interconnected ecosystem within the body, implicates the skin microbiome in the broader chronic disease epidemic, considering the well-established connection between the gut microbiome and most chronic diseases [7].

**Figure 7:**
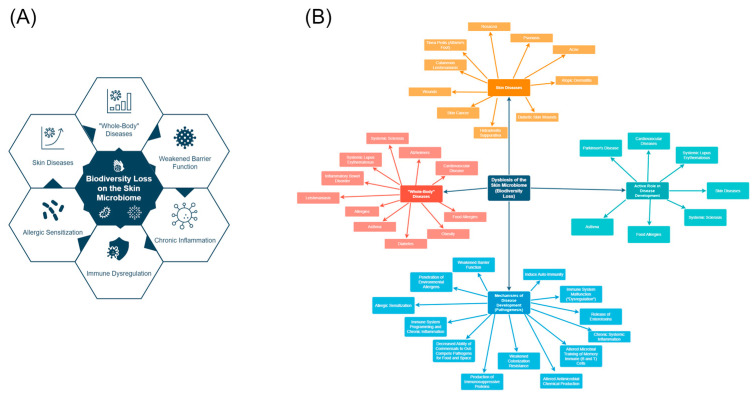
(**A**) shows a simplified version of the potential outcomes associated with biodiversity loss in the skin microbiome. It is important to note that more work needs to be conducted to establish causality and that many outcomes may affect each other. (**B**) shows a map of the diseases associated with reduced biodiversity in the skin microbiome, along with the potential mechanisms of disease development, and finally the diseases in which the literature describes the skin microbiome as potentially playing an active role in their development.

Figure 7 below summarizes the diseases and mechanisms in this section.

## 4. Biodiversity: The Link to Entropy and Ecosystem Health

The UN Convention on Biological Diversity formally defined biodiversity as “the variability among living organisms from all sources, including, inter alia, terrestrial, marine, and other aquatic ecosystems and the ecological complexes of which they are part; this includes diversity within species, between species, and of ecosystems” [213]. It is widely accepted in biology and ecology that high biodiversity corresponds to increased healthiness and functionality within an ecosystem [7,214,215,216,217,218]. It also increases stability, resilience, and invasion resistance [219] and promotes equilibrium [220,221]. The presence of a diverse array of species provides a greater pool of organisms capable of fulfilling the roles necessary to support a healthy ecosystem [55].

While the correlation between biodiversity and ecosystem health is widely accepted by ecologists across various natural ecosystems, including increasingly in the gut microbiome, the same level of certainty has not yet been established for the skin microbiome. Approaching this problem from a physics perspective offers an alternative insight, which is often overlooked, into why biodiversity is crucial for ecosystem health. It could help researchers in the field understand the enormity of the adverse effects caused by biodiversity loss on the skin. Biodiversity is quantified using various indices, such as the popular Shannon Diversity Index, a widely used metric for characterizing biodiversity for decades, represented in Equation (1) below, which is formally identical to the measures of Shannon’s entropy of a system in physics [222]:

(1) $$H=-\sum_{i=1}^{s}p_{i}\log p_{i}$$

where *p* is the probability of finding the species *i*.

- *H* = biodiversity index
- *S* = number of species encountered
- *i* = species
- *p_i_* = *n_i_*/*N* and describes “relative abundance”—the probability that a randomly chosen organism is of the *i*th species
- *n_i_* = total number of organisms of a particular species
- *N* = total number of organisms of all species

This is not a coincidence; entropy is a measure of “disorder” and is used to describe many different systems, including in thermodynamics, ecology, and information theory. Complex systems, if left undisturbed, tend to move towards a state of higher entropy. It describes the distribution of individuals (here individual organisms or microbes on the skin) across different, distinct “states”. In biological applications, “states” are types of organisms, such as species of microbes in the microbiome. Therefore, the Shannon diversity index evaluates not only the “richness” (i.e., the number of “states”, here different types of organisms), but also the “evenness” of an ecosystem (i.e., a measure of the similarity of the abundance of different “states”, here how similar the abundance is between each type of organism) [223]. As a result, the highest biodiversity is predicted when the “spread” between organisms is the greatest [224].

**Figure 8:**
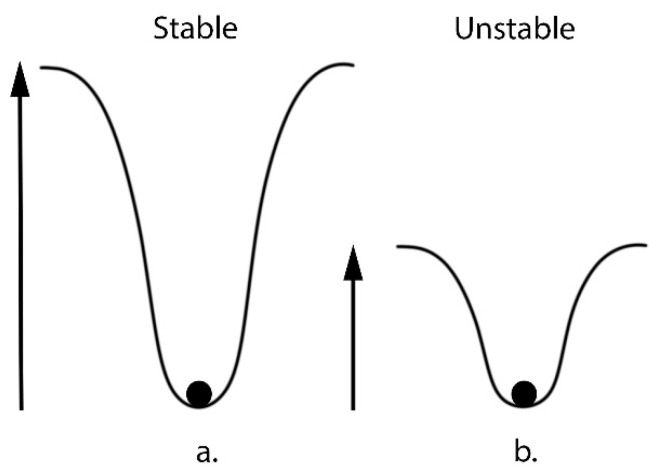
The stable system shown in (**a**) (here representing an ecosystem with increased biodiversity) needs a much larger energy input to move from its current state. Stability refers to the resilience of a system against external factors and changes that impact the system. For the unstable system shown in (**b**) (here representing an ecosystem with reduced biodiversity), a smaller external energy input is needed for it to be able to move from its current state to another state.

Thus, the higher the biodiversity of an ecosystem, the higher the entropy. This relationship also explains the strong correlation between biodiversity and stability in ecological literature. The movement towards the highest entropy state is the most likely outcome, as systems become more stable when their components are spread out in a more disordered state. A stable system exhibits resilience against external influences or changes. To explain this, researchers often use the example of a chemical reaction—the more stable an element is, the more energy is needed to trigger a reaction. An element’s stability is determined by the “potential well” in which its electrons reside. The higher the potential well, the more energy is needed for the element to undergo a reaction (Figure 8). Relating this back to ecosystems, this implies that the bigger the biodiversity increase, the more stable they could become, and the more “interference” is needed to destabilize the system towards dysbiosis. The link between entropy and ecosystem stability is often discussed in the literature [225]. Thus, it implies that a decrease in biodiversity and therefore entropy may decrease stability and resilience, potentially leading to dysbiotic outcomes.

### How Biodiversity Loss Negatively Affects Ecosystems

A decrease in biodiversity can have profound negative repercussions on both macro- and micro-ecosystems, undermining their functionality, efficiency, and capacity to sustain a healthy environment [23,216,226]. It also causes a lack of resilience and stability and affects the ecosystem’s ability to rebuild and rebalance after adverse events [227].

Such a decline also diminishes an ecosystem’s resilience in the face of environmental changes [221]. Once the delicate balance is disturbed and biodiversity diminishes, detrimental effects affect various organisms and can manifest themselves in numerous ways. For example, human interventions in macro-ecosystems can impair the ability of land to perform crucial functions [17].

Biodiversity loss in macro-ecosystems can affect humans, especially those in poorer parts of the world. It can decrease protection against infectious diseases [228], reduce crop yields [229,230], and reduce access to good-quality air and water [231].

## 5. How Do We Regain the Lost Biodiversity?

If biodiversity loss within the human microbiome proves to be a significant factor in the chronic disease epidemic, it may become imperative to explore potential solutions for restoring biodiversity to levels observed in our ancestors.

Similar to the gut microbiome, the use of topical probiotics holds the potential to revolutionize therapeutic treatments, but research has not been fully conclusive [7,232,233]. Although positive study outcomes have been observed [234,235,236], cautionary findings indicate that incorrect implementation of probiotics could lead to damage and reduced biodiversity [232,237], especially for the immunocompromised [238]. It is currently thought to be difficult to meet criteria for safe and effective use of probiotics, and solutions may accidentally introduce incorrect numbers and potentially harmful non-native species [232]. An idea to remedy this is the introduction of “bacterial predators” that are correctly identified and introduced in precise amounts [7,239], mirroring the success of the re-introduction of the wolves to Yellowstone Park [240].

Just as our ancestors enriched soils in the Amazon rainforest to aid plant growth [241], a potential path is to cultivate optimal conditions for skin biodiversity to thrive. Initial steps could entail minimizing exposure to harmful Western environmental factors (Section 2) and recreating the skin’s natural environment through techniques like pH and electrolyte balance [11,110,242,243].

Pre- and postbiotics, inspired by studies on the gut microbiome, require further investigation for their application on the skin. Both have great potential, and neither deals with the risks of applying live microbes, but because they do not colonize the skin, their transient effects may need to be topped up [244].

Given the gut-skin axis, simultaneous restoration and rebuilding of the gut and skin microbiomes may exert a more profound influence on overall health than the sum of their individual effects [28,29,30,31]. Diet and exercise are known to modulate the gut microbiota, which could apply to the skin too [245]. Adopting a “whole-body” approach that addresses various aspects of health could yield the most promising outcomes in restoring lost biodiversity and safeguarding against chronic diseases.

## 6. Future Perspectives: Non-Linearity in Ecosystems and the Butterfly Effect

### 6.1. Could the Non-Linearity of Ecosystems Mean We Have Underestimated the Negative Effects of Biodiversity Loss?

The literature highlights the alarming negative effects of biodiversity loss, both in the human microbiome and macro-ecosystems. However, approaching it from a physics perspective could indicate that we may be prone to underestimating the adverse effects.

This is due to the dependence of complex systems on non-linear physics principles [246]. Very simply, linear systems can be described by simple graphs with a constant gradient and predictable outcomes, which are only altered in proportion to the size of variations in the initial conditions (Appendix A).

In contrast, non-linear systems are represented by equations with variable gradients. The outcomes are disproportionately affected by small changes in the initial conditions. This effect can become even more pronounced when applied to a complex system of millions of interacting components. Nearly every complex system in nature displays non-linearity and, therefore, can behave in ways that are sometimes impossible to predict.

Therefore, could small changes in the biodiversity of the skin have widespread effects on the overall health of the human body? This could further implicate the skin as an important part of the body’s interlinked ecosystem and, as a result, the chronic disease epidemic.

A high-profile example of this phenomenon in an ecosystem is the re-introduction of the wolves to Yellowstone Park, which has been linked to dramatic and deep-rooted changes in the landscape that transformed the health of the system [240]. Just as described by extreme non-linearity, many of the changes were not thought to be possible; rivers changed direction, animal populations rebalanced, plants flourished, and animal habitats were restored. The addition of just one species is a seemingly small change compared to the trillions of interactions within the ecosystem.

### 6.2. Could Chaos Theory and the Butterfly Effect Mean We Have Underestimated the Interconnectedness of the Human Microbiome and Macro-Ecosystems?

The human-caused loss of biodiversity in macro-ecosystems is increasingly associated with the rising disease burden, in part through its impact on the human microbiome [247].

But could this effect go the other way? Could increasing the biodiversity of the human microbiome benefit not just our personal health but also the health of global macro-ecosystems? Despite our efforts to separate ourselves from the global ecosystem, humans and our coexisting microbes are thought to be integral components, as postulated by the Gaia hypothesis [248].

As we have discussed, even tiny changes can disrupt the delicate balance of the ecosystem, potentially leading to disproportionate effects on its health. Complex systems of millions of interacting components can exhibit extreme manifestations of this “non-linearity”, often referred to as “chaos”. Appendix A provides an example of this. This is included in a branch of mathematics called “Chaos Theory”, which describes how complex, dynamical systems are so sensitive that even small variances in the initial conditions could produce widespread and unpredictable changes in the system’s outcomes [249]. It was launched into the mainstream in 1972 by the professor of meteorology at Massachusetts Institute of Technology, Edward Lorenz, when he asked, “Does the flap of a butterfly’s wings in Brazil set off a tornado in Texas?” [250].

Therefore, could the large biodiversity loss seen in the microbiome of humans in the developed world be having a more substantial influence on global ecosystems than currently recognized? Despite our seemingly miniscule role within a vast system, could the daily actions we undertake for our health have larger implications for global health than is currently believed?

## 7. Conclusions

We have shown that there has been a biodiversity loss of up to 84% on the skin of humans in the developed world, mirroring the gut and the ongoing global biodiversity crisis. Chronic diseases are now responsible for 74% of worldwide deaths and afflict 50% of Americans. Moreover, this chronic disease epidemic has already been linked to “mass extinction” in the gut. However, despite being an integral part of the body’s interlinked system, the skin is consistently overlooked in discussions on the topic.

Biodiversity loss on the skin microbiome has been linked to a multitude of skin diseases as well as whole-body diseases. We highlight that biodiversity loss is not only a common finding in dysbiotic ecosystems but also a type of dysbiosis. As a result, we make the case that biodiversity loss on the skin microbiome could be a significant contributor to the chronic disease epidemic. This link between biodiversity loss and dysbiosis is the basis of the paper’s focus on the subject.

Disease development is complex and multifactorial, but research has also shown how dysbiotic skin could play an active role, not only in the development of common chronic skin ailments but in a growing number of whole-body health problems too. These include food allergies, asthma, cardiovascular diseases, Parkinson’s disease, and systemic lupus erythematosus. Damaged skin is now known to cause systemic inflammation, which is thought to be a key driver of many chronic health issues. Other potential mechanisms include the release of toxic proinflammatory chemicals into the bloodstream, immune dysregulation, and allergic sensitization through a weakened barrier.

More evidence for a link between biodiversity loss and chronic diseases could lie in complex physics. We introduce entropy to help evaluate why biodiversity is tied to ecosystem health and stability. Meanwhile, we also introduce ecosystems as being governed by “non-linear physics” principles—including chaos theory—which shows how every individual part of any system is intrinsically linked and implies any disruption to even a small part of the system (here the skin) could have a significant and unknown effect on the overall health of the system (here whole-body health).

Recognizing the inexorable link between ecosystem health and human health allows us to fully understand how crucial it could be to maintain biodiversity across systems everywhere, from the macro-environment we inhabit right down to our body’s microbiome.

## Data Availability

Not applicable.

## Appendix A

A linear system is one that is described by a straight-line graph, meaning it has a constant gradient. It follows a simple equation (or a sum of these equations). Here, x and y are variables, m is the gradient, and c is the constant.

y=mx+c

In contrast, a nonlinear system is one that is not represented by a straight line, has a variable gradient, and is curved. As an example, a general form of a non-linear equation can be:

$$ax^{2}+by^{2}=c$$

It is therefore harder to solve non-linear equations. Nearly every system encountered in nature is non-linear and can behave in ways that make them hard to solve, some even impossible to predict due to an extreme dependency on initial conditions and the large number of components all interacting with each other in different ways.

Issues such as bifurcation and chaos mean that small changes in the initial conditions of a system can cause drastic differences in solutions down the line. This is in contrast to linear systems, where small changes in initial conditions result in proportionately small changes in the solutions. A simple example of an equation that generates chaos is one that describes how the population of an ecosystem of living creatures changes:

$$x_{t+1}=kx_{t}\left(1-x_{t}\right)$$

where $x_{t+1}$ is the population size of the next generation, k is a constant, and $x_{t}$ is the population size now. If one were to “iterate” this equation to see how the population would change, a staggering observation would emerge: abnormal, previously unseen behavior presenting itself as chaos and bifurcation. This behavior seems random but can have a hidden order. The population size is extremely sensitive to initial conditions and unpredictability.
